# Forecasting model of *Grapholita molesta* (Lepidoptera: Tortricidae) in apple orchards

**DOI:** 10.1371/journal.pone.0347667

**Published:** 2026-04-22

**Authors:** Hyunjung Kim, Seonwoong Nah, Hyeon-Ji Yang, DongGeun Choi, Sunghoon Baek

**Affiliations:** 1 Horticulture Division, Jeonbuk State Agricultural Research & Extension Services, Iksan-Si, Jeonbuk, Republic of Korea; 2 Agro-environment Research Institute, Epinet Co., Ltd., Anyang-Si, Gyeonggi, Republic of Korea; 3 Department of Horticulture, Jeonbuk National University, Jeonju-Si, Jeonbuk, Republic of Korea; National Institute of Agricultural Research - INRA, MOROCCO

## Abstract

One of the most notorious pests, *Grapholita molesta*, has caused serious economic damage in apples. However, its phenology model with a defined equation during whole apple crop season has not been developed yet. Therefore, this study was conducted to develop a phenology model of *G. molesta* adult to predict its current and future occurrence patterns. The 1,087 occurrence data sets of *G. molesta* adults from 2013 to 2023 were collected from the Rural Development Administration in Korea. Temperature data of each occurrence data set of *G. molesta* were collected from the Korea Meteorological Administration. The phenology model of *G. molesta* adults were developed with the data sets from 2013 to 2023 with four-peaked Weibull functions. When validated with independent 2024 data, the model developed in this study accurately predicted adult occurrence and reduced prediction errors (in days) for *G. molesta* in Korean commercial apple orchards compared to previous studies. The model predicts that *G. molesta* adults will emerge earlier under climate change scenarios compared to current conditions. In conclusion, this study provides valuable information for controlling *G. molesta* populations in apple orchards.

## Introduction

Apple is the one of most preferred fruits in Korea because it ranks the first in consumed amounts per week [1]. In 2023, apples were cultivated over 34,000 ha accounting for 34% of the total fruit cultivation areas in Korea [2]. Even in sales revenue, the second largest at 25% (1.36 trillion won) after the citrus (1.45 trillion won, 26%) among all fruits in Korea in 2023 [2]. However, apples have been severely damaged by insects.

In Korea, the pest that occurred in apple orchards have been known to 312 species [3]. Even though ten species including *Grapholita molesta*, *Carposina sasakii*, *Panonychus ulmi*, and *Eriosoma lanigerum*, have been causing serious economic damage to apples in Korea [4,5], the oriental fruit moth, *G. molesta*, is an important quarantine pest due to its worldwide invasion [6,7]. This pest invaded America, Europe, and Australia continents [6,7].

The oriental fruit moth overwintered as the late instar stage between barks of trunks closed to the ground [8]. First flight activity of *G. molesta* was mainly observed at early spring when its hosts do not bear fruits. Thus, its progenies feed the new shoots of fruits trees such as peach, apple, pear, plum, apricot, Chinese quince, and so on [8]. As fruits trees start to form their fruits, first instars of *G. molesta* penetrate into the fruits in which the larvae develop with feeding fruit flesh. Until now, the number of flight activities of *G. molesta* has been reported as 4 ~ 5 times [8–17], even though most papers specified its flight activity as four [8–14]. The ecological characteristic of *G. molesta* larvae, feeding and developing within a fruit, makes it difficult to control its populations because most pesticides for its control were not effective when they stay within a fruit [18–21]. Thus, chemical pesticides for this pest are commonly applied more than 10 times during apple crop season in Korea [5]. These frequent applications and low death rate by pesticide application have caused serious a pesticide resistance problem for *G. molesta* control [20,21]. However, its damage could increase up to 71% without suitable management [22–25].

To solve these problems, one of the best solutions is to apply its pesticide at the peak of *G. molesta* larval hatching [18–21]. To define this period, multiple studies [9,13,17,26] reported the peak timing of its adult occurrence. Kim et al. [9] reported 102.3, 628.3, 1,369.9, and 1,876.0 degree days with a lower developmental threshold of 8.1 °C for first, second, third, and fourth occurrence peak, respectively. Damas et al. [13] reported 77.9, 204.8, 578.4, and 1,438 degree days with a lower developmental threshold of 9.5 °C for first, second, third, and fourth peak in peaches, respectively. Both studies [9,13] just estimated its peak timing of each occurrence peak without a specific model, even though the study of Damas et al. [13] used a simple normalized distribution model of the smoothed field observation data. Ma et al. [17] and Amat et. [26] failed to specify the specific peak timing of its occurrence peak. Even though this information is valuable, it is not sufficient for practical applications in agricultural orchards, as the microenvironmental conditions can vary between farms and, consequently, also pesticide applications. Additionally, weather conditions exhibit abrupt and frequent changes; therefore, a model for the flight activities of *G. molesta* is needed to optimize pesticide use. To accommodate these differences and abrupt meteorological changes, a defined model for the flight activities of *G. molesta* should be required.

These meteorological conditions are expected to become more severe [27], which may alter the occurrence patterns of *G. molesta* either over a short period of time or over the entire growing season. In this context, the aim of the current study was to develop a predictive model for the occurrence of *G. molesta* adults throughout the entire apple growing season. Based on the developed model, the change of the occurrence pattern in the future was also predicted.

## Materials & methods

### Occurrence data of *G. molesta* adults

The occurrence data of *G. molesta* adults in apple orchards were obtained from the Rural Development Administration (RDA) of Korea. These data were produced by RDA officers in 167 local RDA centers (S1 Fig). They counted and recorded the attracted adult number every 15 days in the sex-pheromone trap (Delta trap, Green Agrotech Co., Ltd; Gyeongsan-Si, Korea) installed in the commercial apple orchards. The lures and sticky cards (Green Agrotech Co., Ltd) were replaced at one-month and 15-day intervals, respectively. These data were collected from 2013 to 2024. The number of data sets observed during the whole apple crop season was 1,087 from 2013 to 2023. Among them, 549 data sets with at least three non-zero values were used to develop the occurrence model of *G. molesta* adults because one and/or two occurred data could be created by the mistakes of observers. The data sets in 2024 were used to validate the developed model of *G. molesta* adult occurrence in apple orchards of Korea.

### Estimation of cumulative degree-days

To predict the *G. molesta* adult occurrence, the cumulative degree-days (CDDs) were calculated with the single-sine method [28]. The required daily maximum and minimum temperatures for degree-day accumulation were collected from the 510 automated synoptic observing system (ASOS) or the 95 automatic weather system (AWS) of the Korea Meteorological Administration (KMA) which was the closest to each observation site. The degree-day accumulation were started from January 1st with the lower and upper developmental threshold temperature of 8.1 and 32.2 °C suggested by Yang et al. [29] and Li et al. [30], respectively.

### Development of *G. molesta* adult occurrence model

For model development, the data of *G. molesta* adult occurrence in each site were standardized by transforming its occurrence data into the cumulative proportion (dividing the cumulative caught adult number with the total number of adults per year) and by transforming observation dates into CDDs. This standardized data showed four occurrence peaks per year. This occurrence pattern agrees with previous reports describing occurrence models of overwintered populations of *G. molesta* [31], temperature-dependent approaches [32], and oviposition models [33]. Thus, the cumulative occurrence proportions were fitted with a four-peak Weibull function, which was modified from Kim et al. [34] and Baek et al. [35], against the CDDs:


$$f(x)=\frac{\alpha_{\text{1}}}{\text{1}+{\left(\frac{x}{\beta_{\text{1}}}\right)}^{\gamma_{\text{1}}}}+\frac{\alpha_{\text{2}}}{\text{1}+\text{exp}[-\frac{x-(\beta_{\text{1}}+\Delta \beta_{\text{1}})}{\gamma_{\text{2}}}]}+\frac{\alpha_{\text{3}}}{\text{1}+\text{exp}[-\frac{x-(\beta_{\text{1}}+\Delta \beta_{\text{1}}+\Delta \beta_{\text{2}})}{\gamma_{\text{3}}}]}+\frac{\alpha_{\text{4}}}{\text{1}+\text{exp}[-\frac{x-(\beta_{\text{1}}+\Delta \beta_{\text{1}}+\Delta \beta_{\text{2}}+\Delta \beta_{\text{3}})}{\gamma_{\text{4}}}]}$$ (1)

where f(x) is the cumulative occurrence proportions of *G. molesta* adults, x is CDD, $\alpha_{\text{1}}$, $\alpha_{\text{2}}$, $\alpha_{\text{3}}$, and $\alpha_{\text{4}}$ are the parameters representing the relative contribution of the first, second, third, and fourth occurrence peak curves, respectively ($\alpha_{\text{1}}$+ $\alpha_{\text{2}}$ + $\alpha_{\text{3}}$ + $\alpha_{\text{4}}$ = 1), $\gamma_{\text{1}}$, $\gamma_{\text{2}}$, $\gamma_{\text{3}}$, and $\gamma_{\text{4}}$ are the parameters representing the steepness of the first, second, third, and fourth occurrence peak curves, respectively, $\beta_{\text{1}}$ is the time in DDs at 50% occurrence of the first peak curve, $\Delta \beta_{\text{1}}$, $\Delta \beta_{\text{2}}$, and $\Delta \beta_{\text{3}}$ are the times in DDs between the 50% occurrence of the first and second curves, between the 50% occurrence of the second and third curves, and between the 50% occurrence of the third and fourth curves, respectively. The initial parameter estimates were obtained from the distribution plots (S2 Fig) of the occurrence data of *G. molesta* adults. The parameters of this Equation (1) were estimated using PROC NLIN of SAS (SAS Institute Inc.; Cary, NC, USA) [36].

### Model validation of *G. molesta* adult occurrence

Model validation was conducted using 55 data sets, which were not used for model development, of RDA in 2024. In each data set, the end of an occurrence peak was defined as the ending point of the continuous decrease after the continuous increase in *G. molesta* adults. For each occurrence peak, the 50% cumulative occurrence predicted by the model was compared to the observed values, which was estimated via linear regression between the two nearest data points of each RDA data set. For comparison, the CDD values at each peak and site were transformed as the Julian days.

Moreover, the results of the developed model in this study were also compared with the results of Kim et al. [9] and Damas et al. [13] in predicted CDDs of each occurrence peak of *G. molesta*. The 55 data set used in the model validation were used again. The CDDs calculation methods used in these comparisons were same described in the previous section of this study except for the lower developmental threshold, 9.5 °C for the study of Damas et al. [13], and no upper developmental threshold for both studies [9,13]. The deviation of expected and observed 50% cumulative occurrence data according to applied studies were analyzed with a one-way analysis of variance and the mean deviations were separated with a Tukey test using PROC ANOVA [36].

### Future occurrence of *G. molesta* adults

To forecast the adult occurrence of *G. molesta*, the ambient temperature data maps of daily maximum and minimum temperatures under SSP 1–2.6 and SSP 5–8.5 in 2030s, 2050s, 2070s, and 2100s were downloaded from the website of KMA database (http://www.climate.go.kr). The SSP scenarios represent greenhouse gas emission pathways based on future socioeconomic changes, categorized into four pathways (SSP 1, SSP 2, SSP 3, and SSP 5) according to mitigation and adaptation efforts [27]. In this study, we selected SSP1–2.6 (low-emission scenario) and SSP5–8.5 (high-emission scenario) to compare the potential range of climate change impacts on pest phenology, representing the extremes of mitigation efforts and unconstrained emissions. The current (1991–2020) temperature data maps were generated by applying the inverse distance weighting method and topo-climatology model [37] with point temperature data from the KMA database. The CDDs calculation method used in the future distribution was the same described in the previous section of this study using the Python version 3.10 (Python Software Foundation; Wilmington, DE, USA). The used Python code was provided as supporting information (S1 File). The forecasted four occurrence peaks of *G. molesta* adults were visualized with QGIS version 3.16.9 [38].

## Results

**Fig 1:**
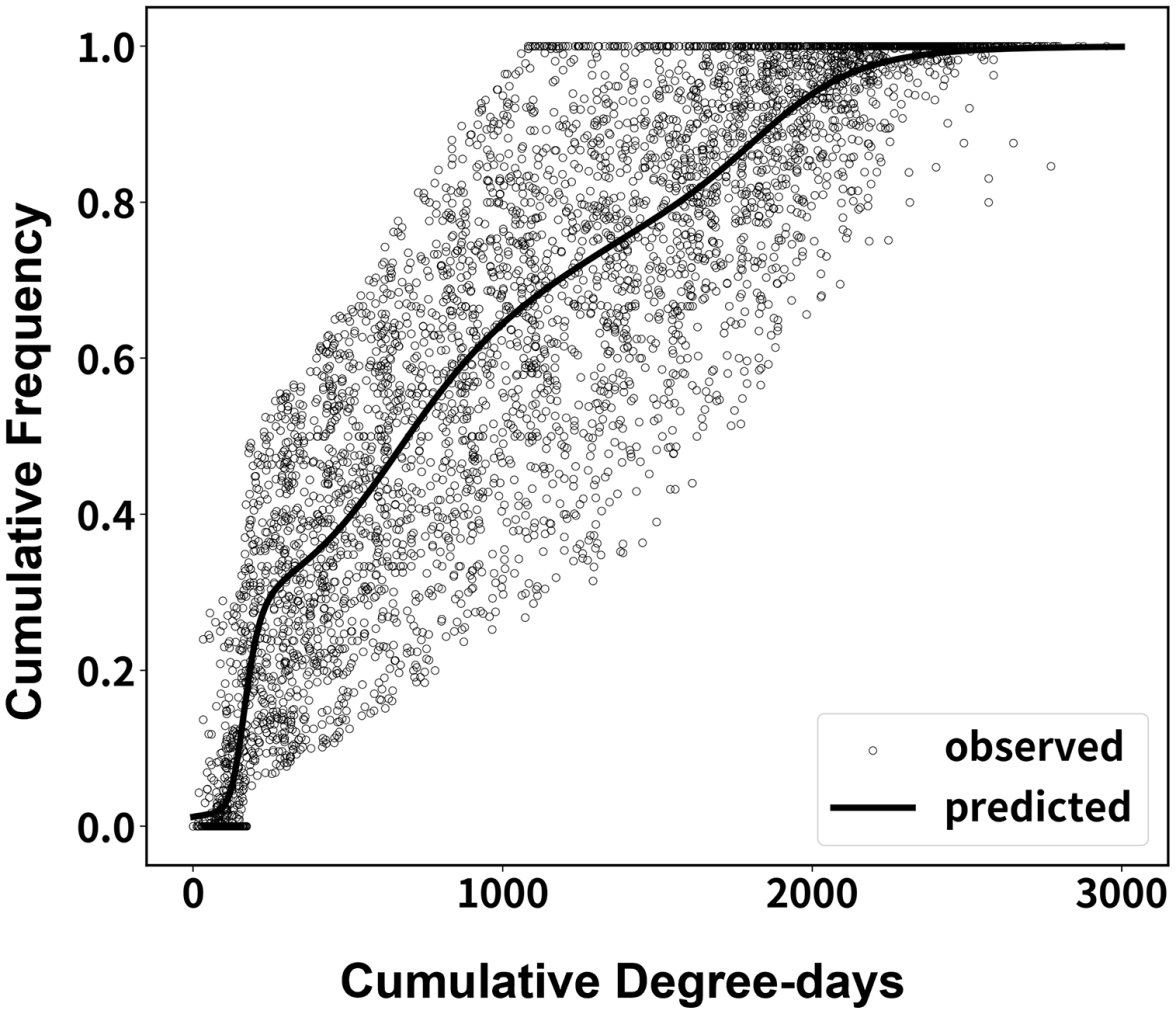
Cumulative observed (2013–2023) and predicted occurrence of *G. molesta* adults in apple orchards in Korea.

The developed model explained well the variations in adult occurrence of *G. molesta* (Table 1, Fig 1; *F* = 2,880.9, df = 11, 5,335, *P* < 0.001, *r*^2^ = 0.86). The first, second, third, and fourth peak roughly occupied 28, 35, 17, and 20% respectively (Table 1). The estimated CDDs of each occurrence peak were 172.2, 636.3, 1,211.6, and 1,816.0 DDs for the first, second, third, and fourth peak, respectively (Table 1, Fig 1). Although the developed model accurately predicted the occurrence of *G. molesta* adults, there was large variability in estimated parameters (Table 1, Fig 1).

**Table 1:** Parameters (estimated values ± SEM) in the developed model for *G. molesta* occurrence in Korean apple orchards.

Parameters	Estimates ± SEM	Parameters	Estimates ± SEM
$\alpha_{\text{1}}$	0.2751 ± 0.0243	$\alpha_{\text{3}}$	0.1724 ± 0.6155
$\beta_{\text{1}}$	172.23 ± 4.0759	$\Delta \beta_{\text{2}}$	575.33 ± 960.47
$\gamma_{\text{1}}$	-7.6149 ± 1.3911	$\gamma_{\text{3}}$	0.0904 ± 0.1663
$\alpha_{\text{2}}$	0.3501 ± 0.4270	$\alpha_{\text{4}}$	0.2024*
$\Delta \beta_{\text{1}}$	464.08 ± 1414.63	$\Delta \beta_{\text{3}}$	604.38 ± 936.29
$\gamma_{\text{2}}$	0.0586 ± 0.0312	$\gamma_{\text{4}}$	0.0604 ± 0.0379

*$\alpha_{\text{4}}$ was estimated from 1 – $\alpha_{\text{1}}$ – $\alpha_{\text{2}}$ – $\alpha_{\text{3}}$.

Even though the developed model in this study showed relatively good predictions in Korea during the whole crop season of apples in 2024, the deviations between predicted and observed dates became high at the first and the fourth peaks of *G. molesta* adult occurrence (Table 2). The prediction accuracy was improved when compared to the approaches suggested by Kim et al. [9] and Damas et al. [13] (Table 2). The newly developed model in this study could decrease approximately 2.8 and 30.5 days in average deviations of *G. molesta* adult occurrence during apple crop season in 2024, respectively (Table 2).

**Table 2:** Differences (days, mean ± SE) between model predictions and actual occurrence data of *G. molesta* adults in 2024 using the model developed in the current study and two previously-developed approaches.

Peaks	This study		Kim et al. [9]		Damas et al. [13]	
	Predicted	Observed	Predicted	Observed	Predicted	Observed
First	119	139	104	139	99	139
Second	169	169	168	169	124	169
Third	205	204	206	204	165	204
Fourth	236	242	242	242	217	242
Average differences	6.75 ± 0.067a		9.50 ± 0.084a		37.25 ± 0.132b	

* Means followed by the same letter within a row are not significantly different (*P* > 0.05, Tukey test).

**Fig 2:**
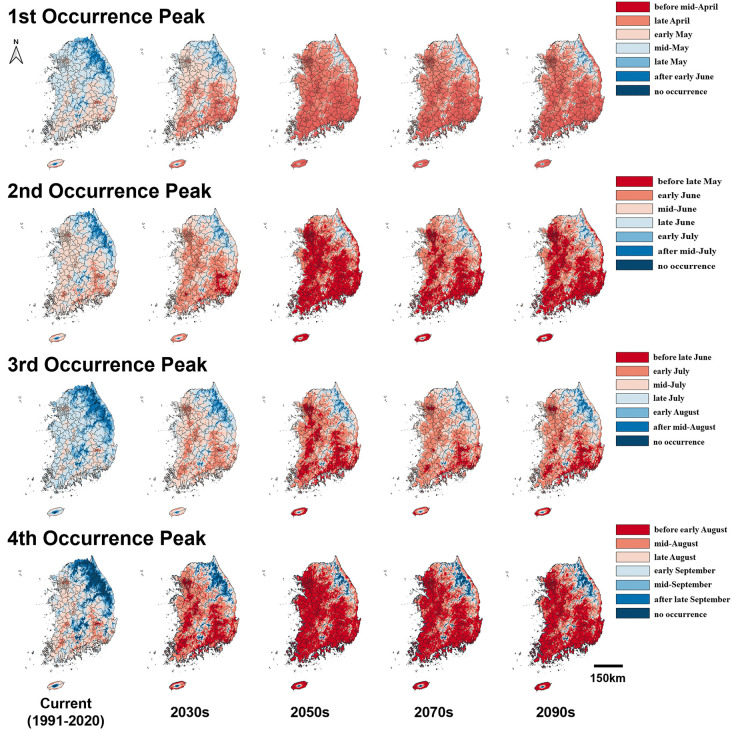
Predicted 50% occurrence timing of *G. molesta* adults in Korea under current (1991–2020) and future (2030s, 2050s, 2070s, and 2100s) SSP 1–2.6 scenarios.

**Fig 3:**
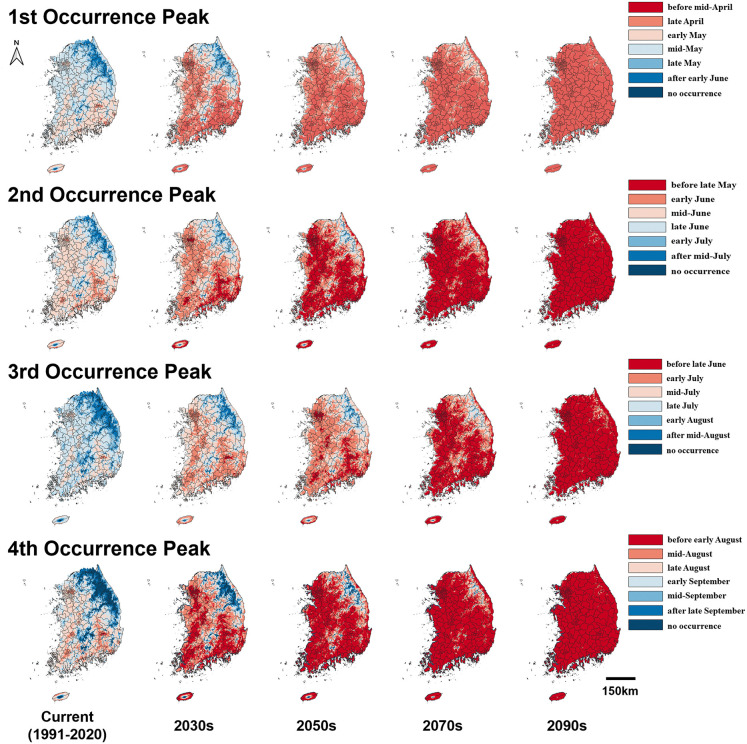
Predicted 50% occurrence timing of *G. molesta* adults in Korea under current (1991–2020) and future (2030s, 2050s, 2070s, and 2100s) SSP 5–8.5 scenarios.

The prediction maps of 50% occurrence timing of *G. molesta* adults based on the average daily min- and max-temperatures 30 years (1991 ~ 2020) showed that its first, second, third, and fourth peaks would mainly occur between the early and late May, the middle of June and early July, the middle of July and the early of August, and the late of August and middle of September, respectively (Figs 2 and 3). Irrespective of the SSP scenarios, its occurrence timing was advanced compared to the current period (Figs 2 and 3). Under SSP 1–2.6 scenario by the 2100s, the first, second, third, and fourth peaks advanced by an average of 23.5, 18.3, 18.8, and 18.5 days, respectively (Fig 2). More pronounced shifts were observed under SSP 5–8.5, with peaks advancing by 50.7, 48.7, 52.1, and 54.5 days on average, respectively. Notably, the shifts in occurrence timing for the second and fourth peaks were more pronounced than those of the first and third peaks, regardless of the SSP scenarios (Figs 2 and 3).

## Discussion

Temperature played a fundamental role in forecasting the occurrence of *G. molesta* adults as other insects [39,40]. The developed phenological model was able to explain the variations in *G. molesta* occurrence and predict their dynamics under field conditions, allowing to forecast the future phenology of *G. molesta* under a climate change scenario.

Clearly, *G. molesta* is multivoltine species. Most entomologists insisted that the adults of *G. molesta* would have four times in their occurrence peaks [8–14], but a few said they had four or five times according to year [15–17]. The voltinism of insects is very important to develop their phenology model because the applied equations to predict their phenology in field conditions should be adjusted to the number of occurrence peaks. The research [15–17] reporting four or five times in their occurrence peaks had a common characteristic, in which the periods of first two peaks included the period of first occurrence peak in those reports [8–12,14] with four peaks in its voltinism. Long occurrence period of overwintering populations in insects is considered as a strategy to increase their survivorship against the unstable environmental conditions during the spring [40,41]. These phenomena have already been reported in several papers [34,35,42]. Especially, Kim et al. [34] reported that *Carposina sasakii*, one of moths in Carposinidae, showed two occurrence peaks for an overwintering generation regardless of year. The five occurrence peaks in *G. molesta* would be caused by the relatively long occurrence period of its overwintering populations. The estimated 464 DDs between first and second occurrence peaks in this study were too short to emerge two generations of *G. molesta*. Thus, modeling four peaks as in this study would be more biologically valid for *G. molesta* than assuming five peaks.

This elongated occurrence pattern of first generation of *G. molesta* would affect its phenology during the whole crop season. The phenology model developed in this study showed that the required degree-days between first and second occurrence peaks (464 DDs) were shorter than those between second and third (575 DDs) and between third and fourth peaks (604 DDs). From the study [32] of Ishiguri, the required degree-days from its eggs to adults were expected to 436.8 DDs. Moreover, the required degree-days for 50% oviposition of its adults were estimated as 149.3 DDs from the study [33] of da Silva et al. Even though these studies [32,33] were conducted in the laboratory conditions, the required degree-days between generations were 586.1 DDs which were similar with the required degree-days between second and third and between third and fourth occurrence peaks in this modeling approach. However, the required degree-days between first and second occurrence peaks in this study were smaller than the estimated theoretical degree-days. This phenomenon might indicate that early occurred individuals of overwintering population (first occurrence peak) would mainly survive and lay eggs to contribute to the first generation (second peak) of *G. molesta* in Korea.

The developed model in this study demonstrated higher accuracy in forecasting *G. molesta* population dynamics in apple orchards than those developed for peaches in previous studies [9,13]. Given that host plants significantly influence insect phenology [40], cross-study comparisons involving different hosts should be interpreted with caution. However, the theoretical degree-days derived from temperature dependent experiments [32,33] suggested that previous phenology models [9,13] were inconsistent with the laboratory findings [32,33]. Moreover, the current study used 549 data sets during 11 years at 167 commercial apple orchards in Korea. Thus, this model developed in this study for *G. molesta* adults would have enough reliability and universality to allow this model to be applied to other field conditions.

However, the model developed in this study is not without its limitation. From its second occurrence peak, some model parameters had high variability with standard errors surpassing the value of the mean; this can lead to uncertainties in the forecasting of its *G. molesta* adults. These phenomena would be primarily attributed to overlapping flight peaks between generations, variations in agricultural practices such as pesticide applications across farms, and sampling errors stemming from observers’ proficiency. These limitations arise from the use of a large dataset consisting of 167 commercial fields over a relatively long period of 11 years. This suggests that while the model accurately reflects general emergence patterns of *G. molesta* adults across diverse field conditions, it exhibits limitations in predicting the relative proportion of each peak and the precise timing of peak occurrence, especially second and third peaks. Therefore, we strongly suggest that the current model undergo pre-adjustment before being utilized for decision-making for developing site-specific models or determining optimal sampling timings for *G. molesta* across diverse conditions.

It is clear that *G. molesta* adults emerge earlier in the future rather than the current according to the climate change scenarios. However, the change would be slower than the expected ones in this study because the emergence of overwintering generations of moths was heavily affected by the day length [35,40,41]. Unless the daylength reaches a critical threshold, moths are unable to terminate diapause, even if temperatures rise progressively. However, rising temperatures would accelerate development, thereby extending adult activity periods. This leads to overlapping flight peaks, reduced peak distinctness, and increased within-season population variability. Consequently, the management windows for *G. molesta* inevitably broadened, making precise applications based on only its phenology model increasingly difficult.

In future emergence prediction of *G. molesta* adults, the first and third occurrence peaks were less dramatically changed compared to ones of second and fourth peaks. The main reason could be explained by that the rate of temperature increases during its first occurrence, spring in Korea, was slower than the one during other occurrence periods of *G. molesta* adults. The slower change of third occurrence peak, summer in Korea, compared to second and fourth ones would be caused by the presence of the upper developmental threshold of *G. molesta*. The hot temperatures more than its upper developmental threshold were frequently expected in SSP scenarios during the summer in Korea.

This study suggested a prediction model of *G. molesta* adult occurrence in apple orchards. The developed model not only accurately predicted actual occurrences under field conditions but also was consistent with theoretical models. Thus, its future occurred pattern based on the developed model in this study would be reliable. These current and future occurrence patterns will be instrumental in optimizing the timing for sampling and management of *G. molesta*.

## Supporting information

S1 Fig Location and distribution of 167 Rural Developmental Administration local centers in Korea. (TIF)

S2 Fig Partial (60 among 532 plots) distribution plots of the occurrence data of *G. molesta* adults in apple orchards in Korea from 2013 to 2023. Name located on the middle top of each plot (e.g., GM-0001-GG-Incheon indicates *G. molesta*-plot number of all plots-abbreviation of province name-city name); The plot features four vertical lines (·). The first peak is situated between the first and second lines, the second peak lies between the second and third, the third peak falls between the third and fourth, and the fourth peak is positioned after the fourth line).
(TIF)

S1 File Code used in this manuscript. (PY)
